# Vascular leakage caused by loss of Akt1 is associated with impaired mural cell coverage

**DOI:** 10.1002/2211-5463.12621

**Published:** 2019-03-20

**Authors:** Jung Min Ha, Seo Yeon Jin, Hye Sun Lee, Farzaneh Vafaeinik, Yoo Jin Jung, Hye Jin Keum, Sang Heon Song, Dong Hyung Lee, Chi Dae Kim, Sun Sik Bae

**Affiliations:** ^1^ Biomedical Research Institute Gene and Cell Therapy Center for Vessel Associated Disease Department of Pharmacology Pusan National University School of Medicine Yangsan Korea; ^2^ Biomedical Research Institute Department of Internal Medicine Pusan National University Hospital Busan Korea; ^3^ Department of Gynecology and Obstetrics Pusan National University Yangsan Hospital Korea

**Keywords:** Akt, angiogenesis, coverage, endothelium, mural cell, vascular leakage

## Abstract

Angiogenesis plays a critical role in embryo development, tissue repair, tumor growth and wound healing. In the present study, we investigated the role of the serine/threonine kinase Akt in angiogenesis. Silencing of Akt1 in human umbilical vein endothelial cells significantly inhibited vascular endothelial growth factor (VEGF)‐induced capillary‐like tube formation. Mice lacking Akt1 exhibited impaired retinal angiogenesis with delayed endothelial cell (EC) proliferation. In addition, VEGF‐induced corneal angiogenesis and tumor development were significantly inhibited in mice lacking Akt1. Loss of Akt1 resulted in reduced angiogenic sprouting, as well as the proliferation of ECs and mural cells. Addition of culture supernatant of vascular smooth muscle cells (VSMCs) in which Akt1 was silenced suppressed tube formation, the stability of preformed tubes and the proliferation of ECs. In addition, attachment of VSMCs to ECs was significantly reduced in cells in which Akt1 was silenced. Mural cell coverage of retinal vasculature was reduced in mice lacking Akt1. Finally, mice lacking Akt1 showed severe retinal hemorrhage compared to the wild‐type. These results suggest that the regulation of EC function and mural cell coverage by Akt1 is important for blood vessel maturation during angiogenesis.

**Abbreviations**
Ang‐1: angiopoietin‐1
EBM: endothelial growth basal medium
EC: endothelial cell
FITC: fluorescein isothiocyanate
HUVEC: shuman umbilical vein endothelial cells
P6: postnatal day 6
P7: postnatal day 7
PDGF: platelet‐derived growth factor
PI3K: phosphatidylinositol 3‐kinase
VEGF: vascular endothelial growth factor
VSMCs: vascular smooth muscle cells

Angiogenesis is the process by which new blood vessels are formed from pre‐existing vessels. This process plays a crucial role in physiological conditions, including wound healing, tissue remodeling and embryonic development, and is also a characteristic of pathological conditions such as tumor angiogenesis, retinopathy of prematurity and diabetic retinopathy 1, 2. Angiogenesis involves the proliferation and migration of endothelial cells (ECs) and stabilization by recruited mural cells such as pericytes and vascular smooth muscle cells (VSMCs) 3. The retinal vasculature is initiated by sprouting from the optic nerve into peripheral regions after birth and is composed of superficial, intermediate and deep layers. In the developing mouse retinal vasculature, tip cells guide capillaries toward a vascular endothelial growth factor (VEGF) gradient and this leads to filopodia formation at the front of the retinal vascular plexus 4. From postnatal day 7 (P7), superficial capillaries start sprouting perpendicularly to form deep layers, which finally form a three‐layered vascular system 5, 6.

Although EC regulation is considered to be important for angiogenesis, interactions between ECs and VSMCs have recently drawn attention. For example, paracrine or autocrine pathways of various ligand‐receptor axis between ECs and smooth muscle cells play essential roles in the regulation of angiogenesis. VEGF binds to their cognate receptor on ECs and regulates EC proliferation and migration 7. Furthermore, platelet‐derived growth factor‐B (PDGF‐B) is secreted by the immature endothelium of growing arteries and regulates mural cell survival, recruitment and proliferation. For example, ablation of PDGF‐B resulted in the loss of mural cells 8. In addition, it has been reported that PDGF‐B and PDGF‐B receptor are involved in the pericyte and VSMC recruitment to growing arteries 9.

Angiopoietin‐1 (Ang‐1) is a paracrine ligand of Tie2 receptor that is highly expressed in ECs and is mainly expressed in pericytes 10, 11, 12. Ang‐1 is known to play a key role in the stabilization of vasculature. For example, Ang‐1 promotes the proliferation and migration of ECs and blocks VEGF‐induced endothelial permeability 13, 14, 15. By contrast, Ang‐2 is secreted by ECs and plays an essential role in angiogenic remodeling by facilitating vessel regression 16 and acts as an antagonist of Ang‐1. These findings suggest that signaling pathways related to Ang‐1 in ECs or pericytes are important for regulating blood vessel stabilization.

Tie2 is classified as a receptor tyrosine kinase 17 and, when occupied by Ang‐1, it activates a series of signaling cascades, including the phosphatidylinositol 3‐kinase (PI3K) and Akt cascades. PI3K has also been reported to be involved in the regulation of angiogenesis. For example, PI3K inhibition impaired retinal angiogenesis in zebrafish 18 and EC‐specific PTEN knockout increased retinal vascular development 19.

The serine/threonine kinase Akt is activated by phosphatidylinositol‐3,4,5‐trisphosphate, which is a product of PI3K 20. Akt has three mammalian isoforms, namely Akt1, Akt2 and Akt3 1, which share more than 80% amino acid sequence homology 21. Nonetheless, these three Akt isoforms have distinctive functions. A deficiency of Akt1 results in growth retardation as a result of defective placental development. Mice lacking Akt2 exhibited insulin resistance and a type 2 diabetes‐like syndrome, whereas brain sizes and weights were reduced in Akt3 knockout mice 1. It has also been reported that Akt activation is important for angiogenesis. For example, silencing of the Akt substrate Girdin abolished VEGF‐induced EC tube formation 22. In addition, selective knockout of Akt1 in ECs resulted in the defect of Akt‐dependent substrate phosphorylation, thereby retarding retinal angiogenesis 23. However, although blood vessel density and sprouting rates were significantly reduced, vascular integrity and context appeared to be normal.

In the present study, we investigated the role of Akt1 in angiogenesis and focused on EC functions, as well as retinal, corneal and tumor angiogenesis, in mice lacking Akt1.

## Materials and methods

### Animals

All of the experimental procedures were performed in accordance with the Animal Care Guidelines of the Laboratory Animal Resource Center of Pusan National University School of Medicine after receiving approval of Pusan National University Institutional Animal Care and Use Committee (mouse: PNU‐2018‐1916 rat: PNU‐2018‐1918). Mice lacking *Akt1* (Akt1^−/−^, B6.129P2‐ *Akt1* ^*tm1Mbb*^/J) were purchased from The Jackson Laboratory (Bar Harbor, ME, USA) and C57BL/6 (wild‐type) mice and rats were purchased from Koatech (Pyeongtaek, Gyeonggi‐do, Korea). Mice were housed under a 12:12 h light/dark cycle at 21–23 °C. Animals were fed normal basal diet (Purina Rodent Chow #38057; Cargill Agri Purina, Seoul, Korea).

### Materials

Endothelial cell culture media were obtained from Lonza, Inc. (Walkersville, MD, USA). Dulbecco's modified Eagle's medium, fetal bovine serum, trypsin‐EDTA and penicillin/streptomycin were purchased from Hyclone Laboratories Inc. (Logan, UT, USA). Anti‐eNOS, anti‐phospho‐eNOS (Ser1177) and anti‐CD31 antibodies were obtained from BD Biosciences (San Jose, CA, USA). Antibodies against pan‐Akt, phospho‐Akt (Ser473) and phosphor‐histone H3 (Ser10) were purchased from Cell Signaling Technology (Boston, MA, USA). Anti‐Akt1, anti‐Akt2 and anti‐neural/glial antigen 2 (NG2) antibodies were purchased from Millipore Bioscience (Temecula, CA, USA). Anti‐actin antibody was obtained from MP Biomedicals (Aurora, OH, USA). Antibody against SM22α was purchased from Abcam (Cambridge, UK). GSL I‐isolectin B4 (IB4) was obtained from Vector Laboratories (Burlingame, CA, USA). Alexa Fluor 488‐conjugated streptavidin, Alexa Fluor 488‐conjugated goat anti‐rabbit, Alexa Fluor 488‐conjugated goat anti‐mouse, Cy3‐conjugated goat anti‐rabbit and Cy3‐conjugated goat anti‐mouse secondary antibodies were purchased from Molecular Probes, Inc. (Carlsbad, CA, USA). Recombinant human VEGF165 was purchased from PeproTech, Inc. (Rocky Hill, NJ, USA). Fluorescein isothiocyanate (FITC)‐dextran (2000 kDa) was purchased from Sigma‐Aldrich (St Louis, MO, USA). IRDye700‐ and IRDye800‐conjugated rabbit and mouse secondary antibodies were obtained from Li‐COR Biosciences (Lincoln, NE, USA).

### Cell isolation and cell culture

Human umbilical vein endothelial cells (HUVECs) were purchased from the American Type Culture Collection (Manassas, VA, USA) and cultured using EGM‐2 MV Bullet kits (Lonza, Inc.) containing 1% penicillin/streptomycin and maintained at 37 °C in 5% CO_2_. VSMCs were isolated from 3‐week‐old Sprague–Dawley rats by a tissue explanting method. The thoracic aorta was isolated and surrounding fat and connective tissues were discarded. Vessels were cut longitudinally and the lumen side was scraped with a razor blade to remove the intima. Vessels were fragmented into lengths of 3–5 mm and explanted lumen side down on collagen‐coated culture dishes. After 7 days of explanting, tissue fragments were discarded and sprouted VSMCs were collected and used (passage 2–3) for the experiments. A precise description of the method is provided in a previous study 24.

### Western blotting and immunocytochemistry

For western blotting, cells were lysed in 20 mm Tris‐HCl (pH 7.4), 1 mm EGTA/EDTA, 1% Triton X‐100, 1 mm Na_3_VO_4_, 10% glycerol, 1 μg·mL^−1^ leupeptin and 1 μg·mL^−1^ aprotinin. After centrifugation, cell lysates were subjected to SDS/PAGE and transferred to nitrocellulose membranes, which were then incubated with indicated primary antibodies and IRDye‐conjugated secondary antibodies. Protein bands were visualized using an infrared image analyzer (Li‐COR Biosciences) 25.

### Lentiviral knockdown

HEK293‐FT packaging cells (Invitrogen, Carlsbad, CA, USA) were grown to approximately 70% confluence in 100‐mm cell culture dishes and triply transfected with 20 μg of pLKO.1 lentiviral vector (control) or containing shAkt1, 5 μg of Δ8.9 and 5 μg of pVSV‐G using the calcium phosphate method. Medium was replaced with fresh medium 8 h after transfection. Lentiviral supernatants were harvested 24 and 48 h after transfection and passed through 0.45‐μm filters. Cell‐free viral culture supernatants were used to infect contractile VSMCs in the presence of 8 μg·mL^−1^ of polybrene (Sigma‐Aldrich). After puromycin selection (10 μg·mL^−1^) for 2 days, at least 95% of cells survived and were used for the experiments. The target sequence was 5′‐cgagtttgagtacctgaagct‐3′ (sh‐Akt1) 25.

### Cell proliferation assay

For measurement of HUVEC proliferation, HUVECs (5 × 10^4^) were plated on six‐well plates and stimulated with culture supernatant of VSMCs silencing Akt1 for 2 days. Cells were fixed with 4% paraformaldehyde and the nuclei were stained with 4′,6‐diamidino‐2‐phenylindole. Images of stained cells were captured with a fluorescence microscope at ×20 magnification and the number of cells was quantified using image j (National Institutes of Health (NIH, Bethesda, MD, USA).

### Isolation of culture supernatant

Vascular smooth muscle cells were cultured in 100‐mm cell dishes until they reached the subconfluent stage and were washed five times with serum‐free medium to remove the serum component. Cells were incubated in 7 mL of serum‐free medium for 48 h. Conditioned media were collected and centrifuged at 324 ***g*** for 5 min to remove cell debris and then filtered through a 0.2‐μm filter.

### Tube formation assay

The tube formation assay was performed as described previously 26. Growth factor‐reduced matrigel (BD Biosciences) was thawed on ice and 300 μL of this was plated into pre‐cooled 24‐well plates and incubated for 30 min at 37 °C to allow polymerization. HUVECs were suspended in 0.2% endothelial growth basal medium (EBM) with or without VEGF (50 ng·mL^−1^) and 5 × 10^4^ cells of HUVECs were added to matrigel‐coated wells. To assess the role of Akt1 in VSMCs, conditioned medium from VSMCs silencing Akt1 was incubated with HUVECs before the initiation of tube formation by EGM or preformed tubes for 12 h at 37 °C. For the VSMC coverage assay, VSMCs silencing Akt1 were infected with lentivirus containing pLL3.7‐GFP vector and 1 × 10^4^ cells were incubated with preformed EC tubes for 6 h. Bright field and fluorescence images were obtained using a fluorescence microscope at ×10 magnification (Axiovert200; Carl Zeiss, Jena, Germany). Tube lengths, the number of branch points and the number of GFP‐positive cells were quantified using image j (National Institutes of Health).

### Whole mount staining of retinas

Mice were anesthetized with an intraperitoneal injection of ketamine and xylazine (80 mg and 10 mg·kg^−1^, respectively) and eyes were isolated from postnatal day 6 and 7‐week‐old mice and euthanized in a CO_2_ chamber. Isolated eyes were fixed with 4% paraformaldehyde for 12 h at 4 °C. Cornea, sclera, lenses and hyaloid vessels were removed and the retinas were blocked and permeabilized in blocking buffer (1% BSA and 0.3% Triton X‐100 in PBS) for 12 h at 4 °C. For immunostaining, IB4 was diluted in PBlec solution (1% Triton X‐100, 1 mm CaCl_2_, 1 mm MnCl_2_ and 1 mm MgCl_2_ in PBS, pH 6.8); other primary antibodies were incubated in retinal blocking buffer overnight at 4 °C. Secondary antibodies were diluted in retinal blocking buffer and incubated for 2 h at room temperature. After four washes in PBS containing 1% Triton X‐100, retinas were flat mounted with anti‐fading reagent (2% *n*‐propylgalate in 80% glycerol/PBS solution) and images were obtained with a confocal microscope (FV1000‐ZDC; Olympus, Japan). Retinal angiogenesis was analyzed by measuring the percentage of angiogenic area per total area, sprouting vessel distance from the optic nerve, the number of tip cells per field and filopodia lengths. Proliferation of retinal ECs and mural cells was quantified by counting of either IB4‐ and pH3‐positive cells (yellow) or pH3‐positive cells (red), which were located next to IB4 staining, respectively. Pericyte coverage was quantified by counting of the number of NG2‐positive cells per 1000 μm vascular length. Quantification was analyzed using image j (National Institutes of Health) 26.

### Aortic sprouting assay

Growth factor‐reduced matrigel was plated into 24‐well plates and incubated for 30 min at 37 °C to allow polymerization. Six‐ to 7‐week‐old wild‐type and Akt1^−/−^ mice were euthanized in a CO_2_ chamber. Thoracic aortas were dissected and the surrounding fat and connective tissues were discarded, cut into 0.8 mm long aortic rings and embedded in matrigel‐coated wells. Aortic rings were stimulated with 0.2% EBM or EGM‐2 medium every 2 days. Images were obtained with a fluorescence microscope at ×5 magnification (Axiovert200; Carl Zeiss). Quantification of the sprouting area was presented as a percentage of control using image j (National institutes of Health).

### Corneal angiogenesis assay

This assay was performed as described previously 27. Seven‐week‐old wild‐type and Akt1^−/−^ mice were anesthetized with chloral hydrate (450 mg·kg^−1^, i.p.). After 10 min, alcaine was dropped into the eye. A corneal micropocket was created with a MVR knife in both eyes. Micropellets of sucralfate (Sigma‐Aldrich) coated with hydron polymer (Sigma‐Aldrich) containing 200 ng of VEGF were then implanted into each pocket approximatly 1 mm from the corneal limbus. Ointment containing antibiotic erythromycin was applied to the eye after implantation to prevent infection. Seven days later, mice were anesthetized with 1–2% inhaled isoflurane, and the eyes were captured using a digital camera. For staining, mice were euthanized in a CO_2_ chamber and the eyes were isolated and fixed with 4% paraformaldehyde for 12 h at 4 °C. Primary antibodies were incubated in blocking buffer (3% BSA in PBS‐ Tween‐20) overnight at 4 °C. Secondary antibodies were diluted in blocking buffer and incubated for 2 h at room temperature. Corneas were flat mounted using anti‐fading reagent, and images were obtained with a confocal microscope (FV1000‐ZDC; Olympus). Quantification of sprouting was assessed by measurement of VEGF‐induced vessel sprouting length.

### Tumor angiogenesis assay

For subcutaneous inoculation, mice were anesthetized with an intraperitoneal injection of ketamine and xylazine and B16‐BL6 melanoma cells (2 × 10^5^) were injected subcutaneously into the backs of 6‐week‐old wild‐type and Akt1^−/−^ mice. Two weeks after tumor inoculation, mice were euthanized in a CO_2_ chamber and tumors were isolated and weighed, photographed using a digital camera.

### FITC‐dextran perfusion

Mice were anesthetized with an intraperitoneal injection of ketamine and xylazine. FITC‐dextran (2000 kDa, 200 μL of 50 mg·mL^−1^ in sterile PBS) was injected into the left ventricle of wild‐type and Akt1^−/−^ mice. Five minutes after injection, mice were euthanized in a CO_2_ chamber and eyes were isolated and fixed with 4% paraformaldehyde for 12 h at 4 °C. Retina images were obtained using a confocal microscope (FV1000‐ZDC; Olympus). Vascular leakage was quantified by counting of the number of FITC‐dextran leakage spot using image j (National Institutes of Health).

### Statistical analysis

The results are expressed as the mean ± SEM of multiple experiments. The unpaired Student's *t*‐test was used to determine the significances of intergroup differences. *P* < 0.05 was considered statistically significant.

## Results

### Effect of Akt1 *in vitro* and *in vivo* angiogenesis

**Figure 1:**
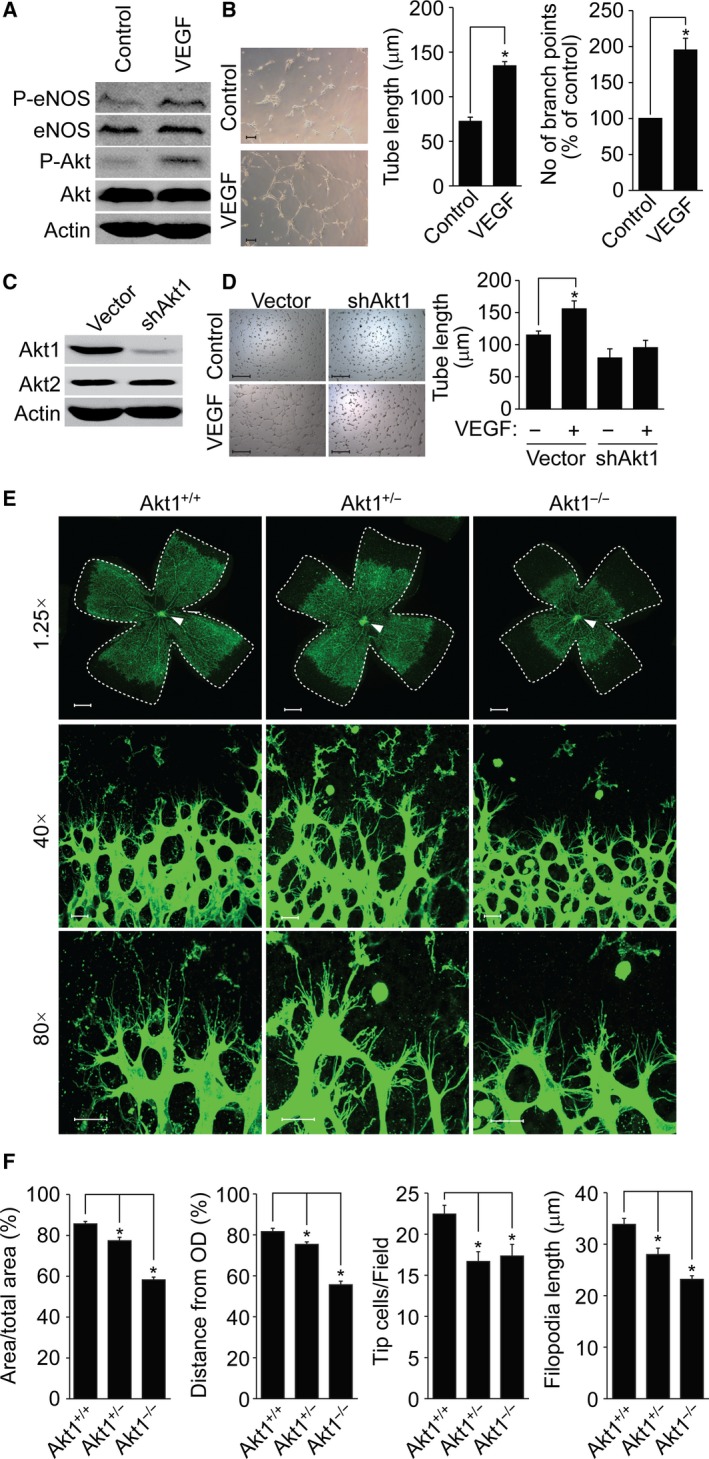
Akt1 regulates angiogenesis *in vitro* and *in vivo*. (A) HUVECs were stimulated with or without VEGF (50 ng·mL^−1^) for 10 min and activation of eNOS and Akt was verified by western blot analysis. (B) VEGF‐dependent tube formation was visualized under a bright‐field microscope at ×10. Scale bar = 100 μm. *P <* *0.05, Student's *‐*test. (C, D) Akt1 expression was silenced in HUVECs and VEGF‐induced tube formation was visualized under a bright‐field microscope at ×10. Scale bar = 500 μm. Tube lengths and the number of branch points were quantified using image j (National Institutes of Health). *P <* *0.05, one‐way ANOVA followed by Tukey's multiple comparison test. (E, F) Retinas isolated from Akt1^+/+^, Akt1^+/−^ and Akt1^−/−^ mice at P6 were stained with IB4 (green) and angiogenesis was analyzed by measuring the angiogenic area, sprouting distance from the optic nerve (indicated by white arrows), tip cell numbers and filopodia lengths using image j (National Institutes of Health). Images were obtained using a confocal microscope at ×1.25 (area), ×40 (tip cells) and ×80 (filopodia; *n* = 4 per group). Scale bar = 800 μm (×1.25) and 50 μm (×40/×80). *P <* *0.05, one‐way ANOVA followed by Tukey's multiple comparison test. Data are the mean ± SEM.

To investigate the role of Akt in angiogenesis, we evaluated EC function after treating HUVECs with VEGF (an angiogenic factor). As shown in Fig. 1A, stimulation of HUVECs with VEGF significantly induced the phosphorylation of eNOS and Akt. In addition, VEGF enhanced capillary‐like tube formation by HUVECs (Fig. 1B). As shown in Fig. 1C,D, VEGF‐induced capillary‐like tube formation was significantly inhibited by silencing Akt1. To confirm the role played by Akt in retinal angiogenesis, we isolated retinas at postnatal day 6 (P6) from Akt1 deficient mice and analyzed its effect on retinal vascular development. As shown in Fig. 1E, outgrowth of superficial retinal vascular plexus was delayed in mice lacking Akt1. In addition, angiogenic area and sprouting distance from the optic nerve were significantly impaired, and tip cell numbers and filopodia lengths were significantly reduced, in the retinas of mice lacking Akt1 (Fig. 1F).

### Effect of Akt1 in angiogenic model system

**Figure 2:**
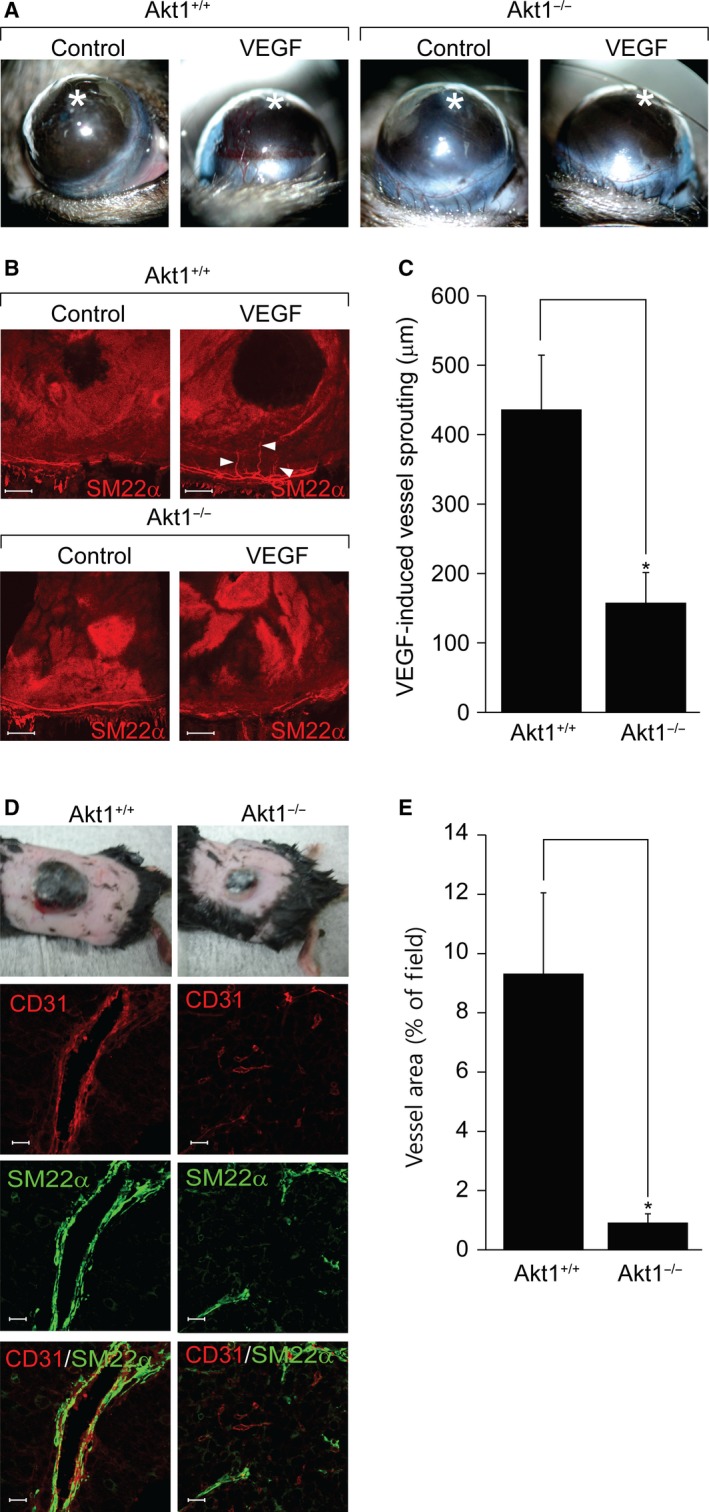
Akt1 is necessary for corneal and tumor angiogenesis. (A, B) A corneal micropocket (indicated by the white star) was created in wild‐type and Akt1 knockout mice and a micropellet containing 200 ng of VEGF was implanted into each corneal pocket. After 10 days of implantation, eyes were imaged using a digital camera and stained with SM22α (red) antibody (*n* = 3 for each group). Arrows indicate newly formed blood vessels. Scale bar = 200 μm. (C) VEGF‐induced vessel length was quantified using image j (National Institutes of Health). *P <* *0.05, Student's *t‐*test. (D, E) Melanoma cells were injected into wild‐type and Akt1 knockout mice and, 2 weeks after injection, tumors were isolated and stained with CD31 (red) and SM22α (green). Images were captured using a confocal microscope at ×60 magnification (*n* = 4 per group). Scale bar = 20 μm. *P <* *0.05, Student's *t‐*test. Data are the mean ± SEM.

We next investigated the role of Akt in various angiogenesis models. As shown in Fig. 2A–C, implantation of VEGF beads into the cornea of wild‐type littermates significantly induced angiogenesis from the cornea limbus to the beads. However, VEGF‐induced vessel sprouting was significantly blunted in mice lacking Akt1. To determine the effect of Akt1 on tumor angiogenesis, we subcutaneously injected melanoma cells into wild‐type littermates and into Akt1 knockout mice. As shown in Fig. 2D,E, tumor volumes and vessel area were significantly reduced in mice lacking Akt1. In addition, vessel structures were smaller and irregular in Akt1 knockout mice.

### Effect of Akt1 in the proliferation of EC and mural cells

**Figure 3:**
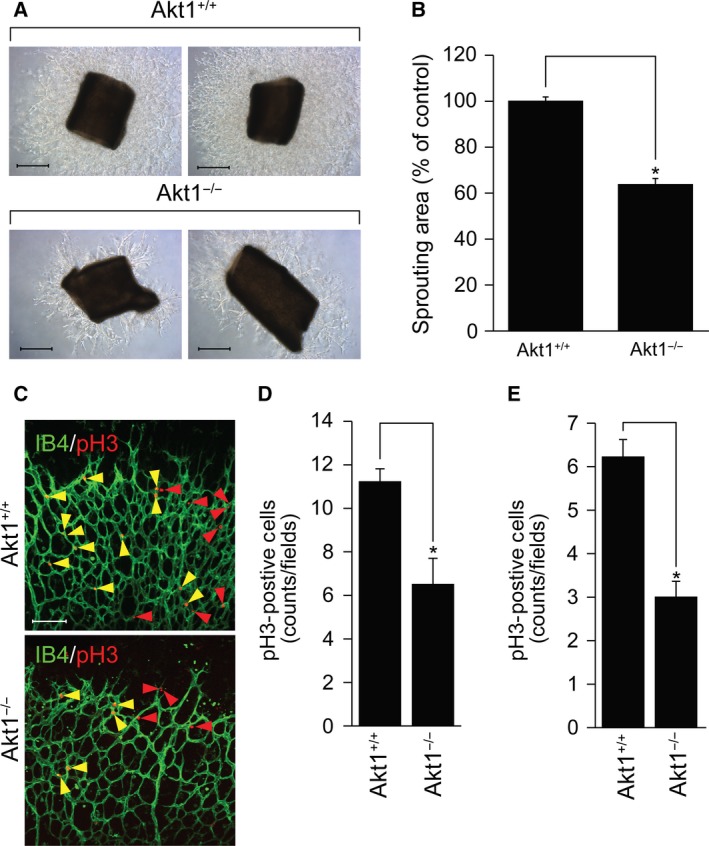
Akt1 regulates proliferation of ECs and mural cells. (A) Aortas were isolated from wild‐type and Akt1 knockout mice and embedded in growth factor‐reduced matrigel‐coated plates in the presence of EGM‐2. After 7 days, bright‐field images were captured under a light microscope at ×5 magnification (*n* = 4 per group). Scale bar = 500 μm. (B) Area of vascular sprouting was quantified using image j (National Institutes of Health) and presented as a percentage of control. *P <* *0.05, Student's *t‐*test. (C) Retinas were isolated from wild‐type and Akt1 deficient mice at P6 and stained with IB4 (green) and pH3 (red). Images were captured on confical microscope at ×20 magnification. Yellow arrowheads indicated pH3‐positive ECs and red arrowheads indicated pH3‐positive mural cells (*n* = 7 per group). The number of pH3‐positive ECs (D) and mural cells (E) was quantified using image j (National Institutes of Health). *P <* *0.05, Student's *t* test. Data are the mean ± SEM.

To investigate the requirement of Akt1 for EC proliferation, we examined EGM‐2‐induced microvessel sprouting in aortic rings isolated from Akt1 knockout mice. As shown in Fig. 3A,B, microvessel sprouting was markedly reduced in aortic rings from mice lacking Akt1. In addition, proliferation of both ECs and mural cells was significantly reduced in retinas from mice lacking Akt1 (Fig. 3C–E).

### Effect of Akt1 in EC‐mural cell communication

**Figure 4:**
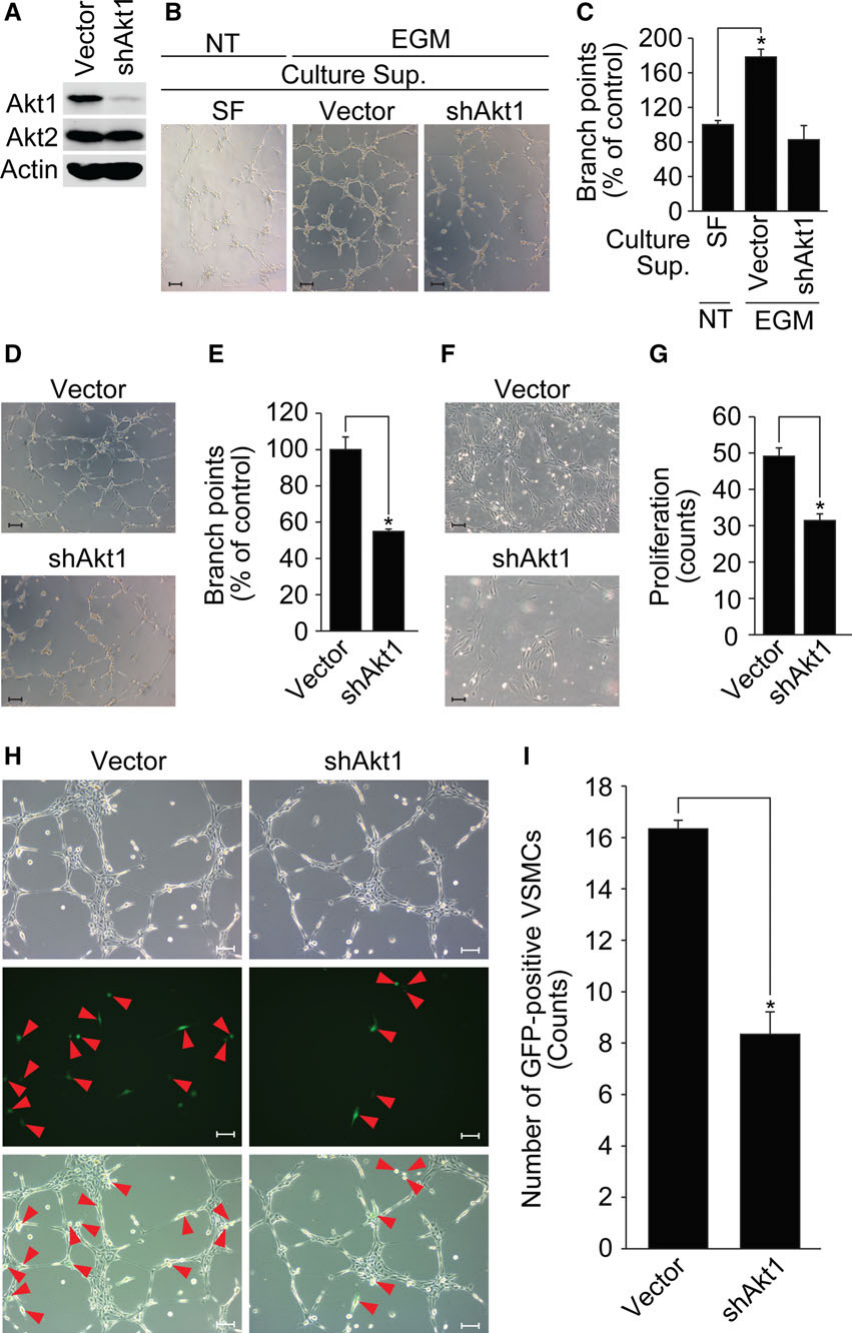
Akt1 in mural cell regulates EC function. (A) Expression of Akt1 was silenced in VSMCs and expression of Akt1/2 was verified by western blot analysis. (B, C) Conditioned medium from VSMCs silencing Akt1 was incubated with HUVECs and EGM‐2‐induced tube formation was verified. Imaged were visualized under a bright‐field microscope at ×10. Scale bar = 100 μm. The number of branch points was quantified using image j (National Institutes of Health). *P <* *0.05, one‐way ANOVA followed by Tukey's multiple comparison test. (D, E) Conditioned medium from VSMCs silencing Akt1 was incubated with preformed EC tubes for 6 h, and images were taken under a bright‐field microscope at ×10. The number of branch points was quantified using image j (National Institutes of Health). *P <* *0.05, Student's *t‐*test. (F, G) HUVECs were stimulated with conditioned medium from VSMCs silencing Akt1 for 2 days and images were captured under a bright‐field microscope at ×10. Scale bar = 100 μm. Proliferation was measured by counting cell numbers. *P <* *0.05, Student's *t‐*test. (H, I) GFP‐tagged VSMCs silencing Akt1 was incubated with preformed EC tubes for 6 h and VSMC coverage was analyzed by counting the number of GFP‐positive VSMCs. Images were visualized under a microscope at ×10. Scale bar = 100 μm. Red arrowheads indicated GFP‐positive VSMCs. *P <* *0.05, Student's *t‐*test. Data are the mean ± SEM.

To confirm EC–mural cell communication, we examined the effect of conditioned medium from VSMCs silencing Akt1. As shown in Fig. 4A–C, tube formation was significantly inhibited in the presence of conditioned medium from VSMCs silencing Akt1. As shown in Fig. 4D,E, incubation of preformed tubes with the conditioned medium from VSMCs silencing Akt1 resulted in the loss of tube‐like structures and disconnected the interaction of individual ECs. In addition, EC proliferation was significantly inhibited by conditioned medium from VSMCs silencing Akt1 (Fig. 4F,G). As shown in Fig. 4H,I, VSMC coverage of EC tube was significantly reduced in cells silencing Akt1.

### Effect of Akt1 in mural cell coverage and hemorrhage

**Figure 5:**
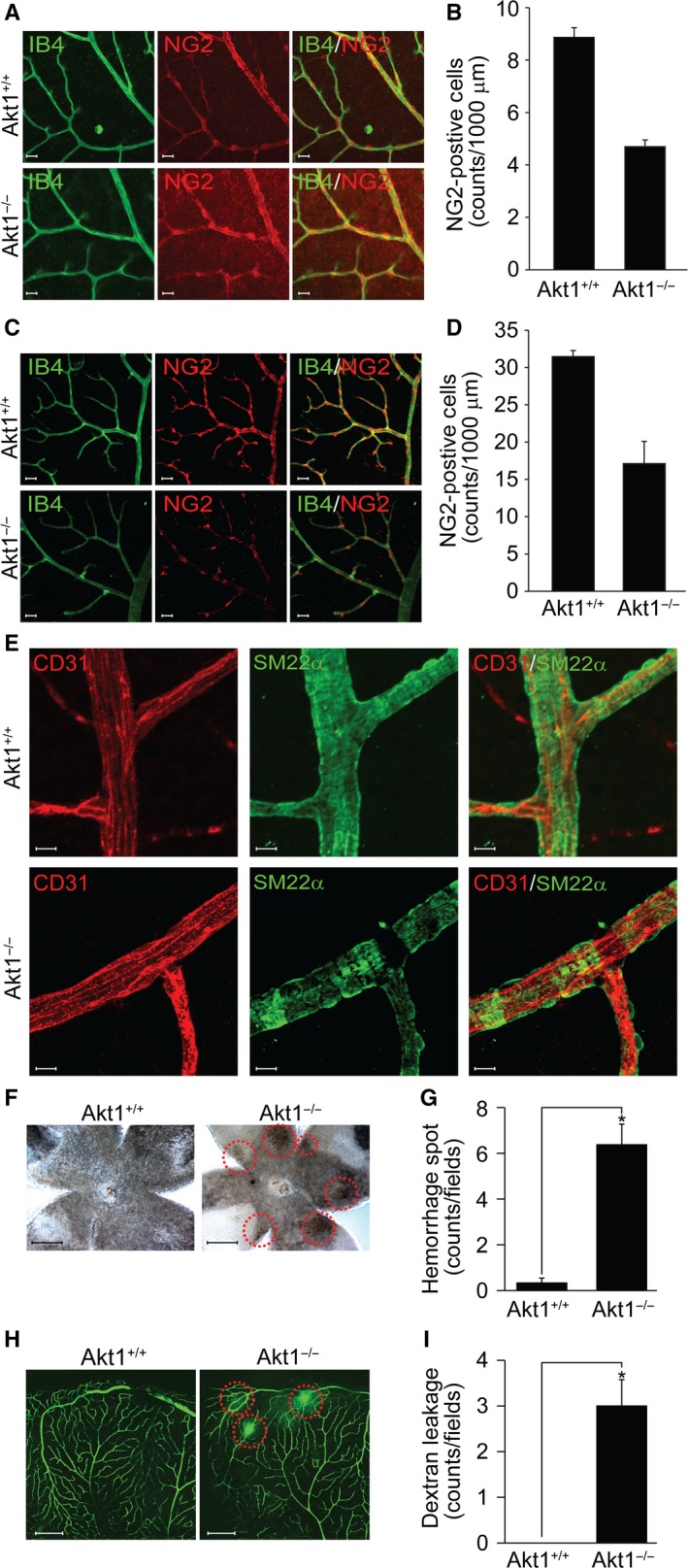
Akt1 regulates mural cell coverage and hemorrhage. Retinas were isolated from P7 (A) and adult (C) mice lacking Akt1 and stained with IB4 (green) and NG2 (red). Images were visualized under a confocal microscope at ×40 magnification (*n* = 4 per group). Scale bar = 100 μm. (B, D) The number of NG2‐positive cells was quantified using image j (National Institutes of Health). *P <* *0.05, Student's *t‐*test. (E) Retinas were stained with CD31 (red) and SM22α (green). Images were visualized under a confocal microscope at ×120 magnification (*n* = 4 per group). Scale bar = 100 μm. (F, G) Retinal vessel hemorrhage was visualized under a bright‐field microscope at ×10 magnification (*n* = 3 per group). Scale bar = 100 μm. The number of hemorrhage spots was quantified using image j (National Institutes of Health). *P <* *0.05, Student's *t‐*test. (H, I) FITC‐dextran was injected into left ventricles and, 5 min later, retinas were isolated and images were captured under a confocal microscope at ×10 magnification (*n* = 3 per group). Scale bar = 200 μm. The number of FITC‐dextran leakage spot was quantified using image j (National Institutes of Health). *P <* *0.05, Student's *t‐*test. Data are the mean ± SEM.

To determine mural cell coverage at the endothelium, retinas were isolated from mice at P7 and also from adult mice lacking Akt1. As shown in Fig. 5, coverage of pericytes (NG2) was significantly reduced in P7 (Fig. 5A,B) and adult (Fig. 5C,D) mice lacking Akt1. VSMCs fully covered large vessels of wild‐type littermates, whereas coverage by VSMCs was incomplete and denuded portions of large vessels were observed in mice lacking Akt1 (Fig. 5E). Because Akt1 null mice showed incomplete blood vessel stability, we next examined vascular leakage. As shown in Fig. 5F,G, retinal vessel hemorrhage was observed in 8‐week‐old mice lacking Akt1. In addition, injection of FITC‐dextran into left ventricles showed severe vascular leakage in mice lacking Akt1 (Fig. 5H,I).

## Discussion

The present study provides *in vitro*,* ex vivo* and *in vivo* genetic data indicating the participation of Akt1 in angiogenesis and vasculature stabilization. First, silencing of Akt1 significantly reduced capillary‐like tube formation by ECs (Fig. 1A–D). Second, genetic loss of Akt1 resulted in delayed retinal angiogenesis and concomitant reductions in tip cell numbers and filopodia lengths (Fig. 1E,F). Third, VEGF‐induced corneal and tumor angiogenesis were significantly attenuated in mice lacking Akt1 (Fig. 2). Fourth, *ex vivo* data showed that sprouting of ECs from aortic tissues isolated from mice lacking Akt1 was markedly reduced (Fig. 3A,B). In addition, proliferation of ECs and mural cells was significantly reduced in Akt1 deficient mice (Fig. 3C–E). Furthermore, conditioned medium from VSMCs silencing Akt1 suppressed tube formation, EC proliferation and preformed tube stability. Finally, coverage of mural cell was significantly reduced in mice lacking Akt1 (Fig. 5A–D). In addition, SM22α‐positive VSMC coverage was attenuated in Akt1 deficient mice (Fig. 5E–I). These observations indicate that the expression of Akt1 appears to be necessary for physiological and pathological angiogenesis. Indeed, several previous studies have also indicated that Akt1 in ECs is required for angiogenesis. For example, Akt1 plays an essential role in mediating VEGF and Ang‐1 signaling cascades, which are important for angiogenesis 28, 29. Currently, the physiological role of Akt1 in ECs has been reported in many aspects. In particular, it is possible Akt1 regulates polarized tip cell movement during angiogenesis. For example, we observed that loss of Akt1 diminished filopodia formation by tip cells (Fig. 1E). In addition, other studies have also shown that Akt1 signaling cascade disruption blocks the chemotactic migration of ECs 30, 31 and it has been reported that endothelial progenitor cell recruitment to angiogenic foci was reduced in mice lacking Akt1 28. Akt1 also regulates cancer and fibroblast cell chemotaxis by lysophosphatidic acid and PDGF 32, 33 and therefore the regulation of tip cell polarization and movement to hypoxic regions by Akt1 plays an essential role in the angiogenic process.

The role of Akt1 in blood vessel stability has been investigated using Akt1 whole body knockout mice. For example, loss of Akt1 induces plaque vulnerability during atherosclerosis or shows impaired blood vessel maturation during wound healing 34, 35. It has also been suggested that Akt1 promotes endothelial barrier protection by the regulation of junctional proteins 36. It is still unknown how Akt1 in EC regulates vascular stability, although recent evidence supports the idea that regulation of thrombospondins expression by Akt1 might be the possible link for the regulation of vascular permeability 37. Additional evidence that Akt1 regulates vascular stability has also been provided. For example, EC‐specific Akt1 knockout mice instigated the apoptosis of VSMCs via an impairment of Jagged1‐Notch signaling in VSMCs 38. It is notable that VSMC coverage is normal in EC‐specific Akt1 deficient mice but markedly reduced in an Akt2 knockout background. Because Akt1 and Akt2 have high sequence homology and some redundant functions, these results suggest that Akt1 in pericytes or VSMCs may play an important role in vascular stability.

The present study raises an important issue regarding the role played by mural cells during angiogenesis and blood vessel stability. Pericytes are undifferentiated mural cells that cover newly formed endothelial tubes, where they differentiate into VSMCs 10. Most pericytes express CD146, smooth muscle actin, NG2 and notably PDGF‐β receptor 39, and the interaction between pericytes and ECs appears to play an essential role in angiogenesis. For example, co‐culture of pericytes with ECs promoted EC tubulization *in vitro* 40. Because pericytes and ECs are embedded within same sites, it is possible that they interact directly via adhesion molecules. The other possibility is that the cells regulate each other in a paracrine manner. For example, secretion of PDGF‐β by ECs recruits pericytes and enhances differentiation into VSMCs 9. The present study also showed that conditioned medium from VSMCs affected the function of EC in terms of proliferation, tube formation and tube stability (Fig. 4). In addition, loss of Akt1 markedly reduced pericyte coverage of tip and stalk cells (Fig. 5A–D). Many other studies have also implicated the paracrine regulation of ECs and mural cells. For example, sphingosine 1‐phosphate, Ang‐1 and Ang‐2 were reported to act as paracrine factors for pericyte and EC interactions 41, 42, 43, 44. Notably, all of these paracrine factors were shown to induce Akt activation 25, 45, 46, 47 and thus it is possible that interactions between pericytes and ECs are regulated by a variety of paracrine factors and also that Akt mediates a signaling cascade leading to vascular stabilization.

Vascular stability is a critical feature of the pathogenesis of vascular diseases. Although EC function plays an essential role in vascular stability, the results of the presnt study showed that Akt in pericytes or VSMCs also plays an important role. For example, selective loss of Akt1 in ECs retards angiogenesis, although vascular stability is not affected 28. Likewise, EC‐specific Akt1 knockout mice were indifferent with respect to the status of vascular stability 38. However, in the present study, we showed that conditioned medium from VSMCs silencing Akt1 facilitated the disruption of tubular structure of EC (Fig. 4D,E). In addition, Akt1 whole body knockout mice clearly showed impaired pericyte and VSMC coverage (Fig. 5A–E), as well as hemorrhage and leaky properties (Fig. 5F–I). These results may have been caused by insufficient pericyte proliferation (Fig. 3C–E) or coverage of ECs (Fig. 5A–D). Because loss of Ang‐1 impairs vascular remodeling and Ang‐1 overexpression induces hypervascularization and leakage resistant blood vessels 42, 48, it is possible that Akt1 in pericytes or VSMCs may regulate the Ang‐1 signaling cascade. It is possible that impairment of vascular stability of whole body Akt1 knockout animal model might be a result of the loss of Akt1 in many cell types and so it still remains unclear whether the expression of Akt1 in mural cells plays a pivotal role in vascular stability. To resolve this issue, an analysis of mural‐specific Akt1 knockout animal model might be required.

In conclusion, whole body loss of Akt1 stunts the proliferation and activation of ECs, thus blocking retinal, corneal and tumor angiogenesis. Inappropriate coverage and a proliferation of mural cells were observed in mice lacking Akt1. In addition, paracrine factors isolated from VSMCs silencing suppressed EC proliferation, tube formation and tube stability. These results suggest that Akt1 in VSMCs might play a pivotal role in vascular stability. Therefore, unveiling the mechanistic pathway of Akt1‐dependent stabilization of blood vessels could represent a therapeutic target for vascular diseases.

## Conflict of interest

The authors declare no conflict of interest.

## Author contributions

JMH performed experiments. SYJ, HSL, FV, YJJ and HJK assisted with the data analysis. SHS, DHL and CDK provided critical comments on the manuscript. SSB conceived the study and wrote the manuscript.
